# Deciphering Multiplicity of HIV-1C Infection: Transmission of Closely Related Multiple Viral Lineages

**DOI:** 10.1371/journal.pone.0166746

**Published:** 2016-11-28

**Authors:** Vlad Novitsky, Sikhulile Moyo, Rui Wang, Simani Gaseitsiwe, M. Essex

**Affiliations:** 1 Harvard T. H. Chan School of Public Health, Boston, Massachusetts, United States of America; 2 Botswana Harvard AIDS Institute Partnership, Gaborone, Botswana; 3 Division of Medical Virology, Stellenbosch University, Tygerberg, South Africa; 4 Division of Sleep and Circadian Disorders, Brigham and Women’s Hospital, Boston, Massachusetts, United States of America; National and Kapodistrian University of Athens, GREECE

## Abstract

**Background:**

A single viral variant is transmitted in the majority of HIV infections. However, about 20% of heterosexually transmitted HIV infections are caused by multiple viral variants. Detection of transmitted HIV variants is not trivial, as it involves analysis of multiple viral sequences representing intra-host HIV-1 quasispecies.

**Methodology:**

We distinguish two types of multiple virus transmission in HIV infection: (1) HIV transmission from the same source, and (2) transmission from different sources. Viral sequences representing intra-host quasispecies in a longitudinally sampled cohort of 42 individuals with primary HIV-1C infection in Botswana were generated by single-genome amplification and sequencing and spanned the V1C5 region of HIV-1C *env* gp120. The Maximum Likelihood phylogeny and distribution of pairwise raw distances were assessed at each sampling time point (n = 217; 42 patients; median 5 (IQR: 4–6) time points per patient, range 2–12 time points per patient).

**Results:**

Transmission of multiple viral variants from the same source (likely from the partner with established HIV infection) was found in 9 out of 42 individuals (21%; 95 CI 10–37%). HIV super-infection was identified in 2 patients (5%; 95% CI 1–17%) with an estimated rate of 3.9 per 100 person-years. Transmission of multiple viruses combined with HIV super-infection at a later time point was observed in one individual.

**Conclusions:**

Multiple HIV lineages transmitted from the same source produce a monophyletic clade in the inferred phylogenetic tree. Such a clade has transiently distinct sub-clusters in the early stage of HIV infection, and follows a predictable evolutionary pathway. Over time, the gap between initially distinct viral lineages fills in and initially distinct sub-clusters converge. Identification of cases with transmission of multiple viral lineages from the same source needs to be taken into account in cross-sectional estimation of HIV recency in epidemiological and population studies.

## Introduction

The majority of HIV infections are associated with transmission of a single founder virus, with transmission of multiple HIV-1 lineages occurring in about 20% of heterosexual cases [1–9]. Multivariant HIV transmission is higher in men who have sex with men (MSM) and injection drug users, reaching about 30–40% [10–13], although no difference in multiplicity of HIV transmission between modes of HIV transmission was also reported [14]. Multiple HIV lineages could be transmitted at a single encounter, or on multiple occasions over the course of HIV infection. The latter scenario is known as an HIV super-infection [7, 15–25]. In the HPTN 052 study, analysis of transmitted HIV’s helped to distinguish linked and unlined viral transmissions and solidify the study findings [26–29]. The multiplicity of breakthrough HIV infections can be an important outcome in vaccine trials [11].

Identification of the multiplicity of virus transmission in HIV infection is challenging because it requires multiple viral sequences representing intra-host HIV quasispecies. Molecular techniques, such as single-genome amplification and sequencing, or next-generation sequencing, can be applied to address multiplicity of HIV transmission, which remains a subject of special studies.

The term *multiplicity* of virus transmission in the context of HIV infection has not been well defined with the exception of two extreme scenarios, transmission of a single founder virus and HIV super-infection. High homogeneity of viral quasispecies soon after infection is associated with the effective transmission of a single HIV variant (which does not exclude transmission of multiple but undetected, or extinguished, variants). Similarly, distinct viral lineages separated by other patients’ sequences in the phylogenetic tree provide compelling evidence for transmission of multiple HIV variants, often as a super-infection. However, the interpretation of intermediate scenarios remains uncertain, as well as thresholds and criteria for multiplicity of HIV transmission. Technically, even a single nucleotide difference between identified intra-host HIV quasispecies could be interpreted as transmission of *multiple* viral variants. However, the clinical or epidemiological relevance of transmitted HIV quasispecies with minor differences is still unclear.

Multivariant HIV infection has been associated with elevated HIV-1 RNA set point [22, 25, 30–33] and faster disease progression [34–37], but has been reported to have limited impact on the occurrence of clinical events [22].

In this study we focus on transmission of multiple HIV lineages from the same source. The goal of the study was to identify transmission of multiple virus lineages from the same source based on the inferred phylogeny and distribution of viral pairwise distances of viral sequences representing intra-host HIV quasispecies. Better understanding of HIV transmission and the ability to distinguish between transmissions of multiple virus variants from a single source and those from multiple sources should assist in the analysis of HIV transmission networks and their dynamics.

## Materials and Methods

### Ethics statement

The study on primary HIV-1C infection in Botswana, the *Tshedimoso* study [4, 38–45], was conducted according to the principles expressed in the Declaration of Helsinki. The study was approved by the Health Research and Development Committee (HRDC) of the Republic of Botswana, and the Office of Human Research Administration (OHRA) of the Harvard T.H. Chan School of Public Health. All adult study subjects provided written informed consent for participation in the study; all minor study subjects provided written informed assent, and each minor’s guardian provided written informed consent, for their participation in the study.

### HIV-1C sequences

Viral sequences were generated within the *Tshedimoso* study of primary HIV-1C infection in Botswana [4, 38–45]. Briefly, 42 individuals with primary HIV-1C infection (including 8 acute and 34 recent cases) were longitudinally sampled over a period of about 500 days post-seroconversion. Viral sequences were generated by single-genome amplification and sequencing and spanned the V1C5 region of HIV-1C *env* gp120, about 1,200 bp in length (HXB2 nucleotide positions 6,615 to 7,757). The initial set of viral sequences included 225 time points; eight time points with fewer than four sequences each were excluded (16 sequences total). The total number of 217 time points analyzed in this study represented 42 patients, median 5 (IQR: 4–6) time points per patient, range from 2 to 12 time points per patient. The analyzed time points were represented by a total of 2,524 sequences, approximately 12 sequences per patient per time point. Participant characteristics are described elsewhere [40, 43, 44]. All participants were infected with HIV-1C, and were predominantly female (76%), with a median age of 27 (IQR 25–33) years at enrollment. Both viral RNA and proviral DNA were used as templates for amplification and sequencing. The GenBank accession numbers of the viral sequences used in this study are KC628761–KC630726 and KX644184–KX644757.

### Multiple sequence alignment

Codon-based multiple sequence alignment of viral sequences was performed by Muscle [46, 47] with default setting for gap penalty and gap extension. Minor manual adjustments across the multiple sequence alignment were performed in BioEdit [48].

### Phylogenetic analysis

The Maximum Likelihood (ML) phylogeny was reconstructed for each time point of sampling (n = 217; 42 patients; median 5 (IQR: 4–6) time points per patient, range from 2 to 12 time points per patient) using PhyML [49, 50]. The resulting phylogeny was visualized in SeaView [51].

### Pairwise distances

The distribution of pairwise raw distances of viral sequences per subject per time point was analyzed by dist.dna (ape [52] package in R) using multiple sequence nucleotide alignment.

### Testing for sub-clusters

The goal of this analysis was to identify (or reject) the presence of potential sub-clusters within each set of viral sequences representing intra-host HIV-1C quasispecies at a single time point. In this paper the term “sub-clusters” indicates clusters within the pool of HIV sequences representing intra-patient viral quasispecies. Sub-clusters were defined by a specific topology in the inferred ML phylogenetic tree: presence of a monophyletic patient-specific lineage with sub-clusters, which was evident by a combination of relatively long branches separating sub-clusters of viral sequences and short branches within each cluster. Such a topology was considered to be associated with transmission of multiple viral variants from the same (or a closely related) source of presumably established (chronic) HIV infection.

To standardize identification of sub-clusters based on phylogeny of viral sequences representing intra-host HIV-1C quasispecies, we developed a simple test using R packages ape [52] and stats [53]. The pairwise distance matrix was generated by dist.dna (ape [52] R package). To identify (or reject) potential sub-clusters, kmeans (stat R package) was utilized to partition the pairwise distance matrix into two groups (k = 2). The ratio of withinss (vector of within-cluster sum of squares, one per cluster) to betweenss (the between-cluster sum of squares) was used to determine the validity of partitioning. The clustering was considered valid if the ratio values (withinss to betweenss) for both sub-clusters were greater than zero and less than a particular threshold. To inform the choice of the threshold, we performed simulation studies to calculate the sensitivity, specificity and predictive values for the ratio withinss to betweenss thresholds set at 0.1, 0.15, 0.20, 0.25 and 0.30 using the R package caret [54]. The clustering estimates at different ratio thresholds were compared with the reference data. The reference data with and without sub-clusters were generated by evaluation of ML phylogeny and distribution of pairwise distances for 217 time points. For each threshold examined, sensitivity was defined as the proportion of clustered cases with this threshold out of the number of clustered cases in the reference data. Specificity was defined as the proportion of non-clustered cases with the specified threshold out of the number of non-clustered cases in the reference data. Positive predictive value was defined as the proportion of predicted time points with sub-clusters out of clustered reference data: sensitivity * Prevalence)/((sensitivity*Prevalence) + ((1-specificity)*(1-Prevalence))). Negative predictive value was defined as the proportion of non-clustered time points out of non-clustered reference data: (specificity * (1-Prevalence))/(((1-sensitivity)*Prevalence) + ((specificity)*(1-Prevalence))). The sensitivity and specificity of different values that were estimated from the simulation studies are presented in Table 1.

**Table 1:** Simulation studies for the ratio withinss to betweenss threshold values.

Threshold values	Sensitivity	Specificity	Positive predictive value	Negative predictive value
0.10	0.989	0.667	0.949	0.909
0.15	0.989	0.700	0.954	0.913
0.20	0.977	0.767	0.963	0.852
0.25	0.947	0.767	0.962	0.697
0.30	0.909	0.767	0.960	0.575

Based on the results of simulation studies, the value of 0.20 has been chosen as the threshold for ratio withinss to betweenss, although the value of 0.15 could also be considered. In this study, within each set of viral sequences representing intra-host HIV-1C quasispecies at a given time point, sub-clusters were considered present if the ratio values for both sub-clusters were greater than zero and less than 0.20.

### Statistical analysis

The statistical analysis was performed in R version 3.3.1 [53]. The proportions and the associated 95% confidence intervals (CIs) of transmitted multiple viral variants were estimated based on binomial distributions (prop.test() in R). McNemar’s test [55] was used to compare the proportions of two dichotomous traits from the same group of subjects. The rate of HIV super-infection was estimated by using the participants’ maximum follow-up time and was expressed as the number of events per 100 person-years. P-values less than 0.05 were considered statistically significant. The reported p-values are 2-sided.

## Results

HIV-1C evolutionary dynamics are exemplified by the following four scenarios: (1) transmission of a single founder virus, (2) transmission of multiple viruses from the same source partner, (3) super-infection, and (4) transmission of two founder viruses followed by an HIV super-infection. The inferred ML trees and distribution of pairwise distances are presented at the specified time points of sampling expressed as days post-seroconversion.

### Transmission of a single founder virus

**Fig 1:**
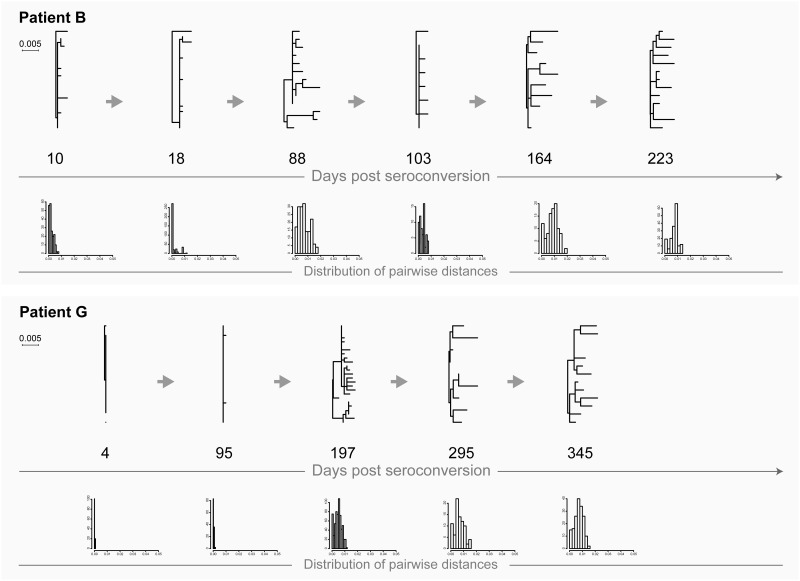
HIV transmission of single viral variants in acutely infected patients B and G. Maximum likelihood trees inferred by PhyML and distribution of raw pairwise distances are shown. Numbers below each phylogenetic tree indicate time of sampling in days post-seroconversion. Both phylogenetic trees and histograms of pairwise distances are drawn to the patient-specific scale shown at left for each patient.

**Fig 2:**
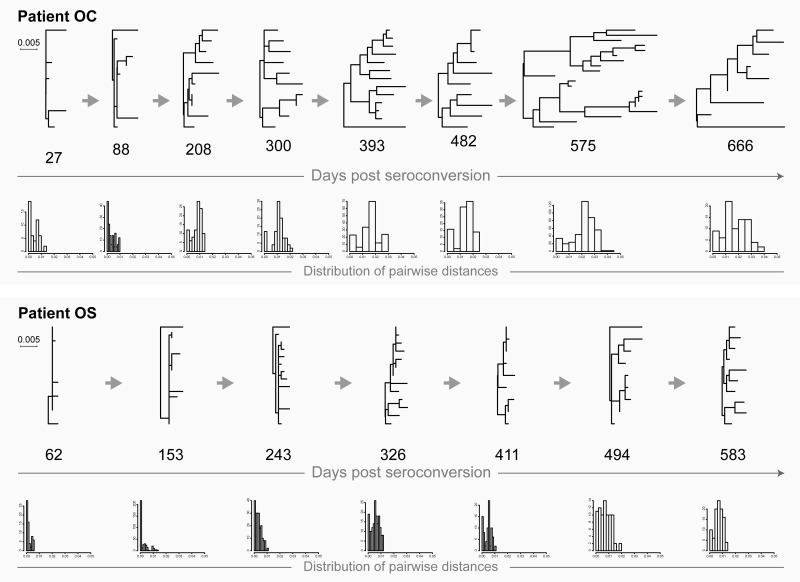
HIV transmission of single viral variants in recently infected patients OC and OS. For explanation of phylogenetic trees, pairwise distances, and time points of sampling, please see Fig 1 legend.

Extreme homogeneity of viral quasispecies in most cases is a hallmark of transmission of a single viral variant (Fig 1: patients B and G; Fig 2: patients OC and OS). It is possible that homogeneity of viral quasispecies could also occur during transmission of multiple viruses followed by a single variant outgrowth. Viral diversity increases gradually over time (with or without fluctuations) which is evident from the increasing branch lengths in the phylogenetic tree. The close to normal distribution of pairwise distances reflects gradual increase of viral diversity over time. The majority of HIV infections follow this scenario.

### Transmission of multiple (i.e., two) viruses from the same source

**Fig 3:**
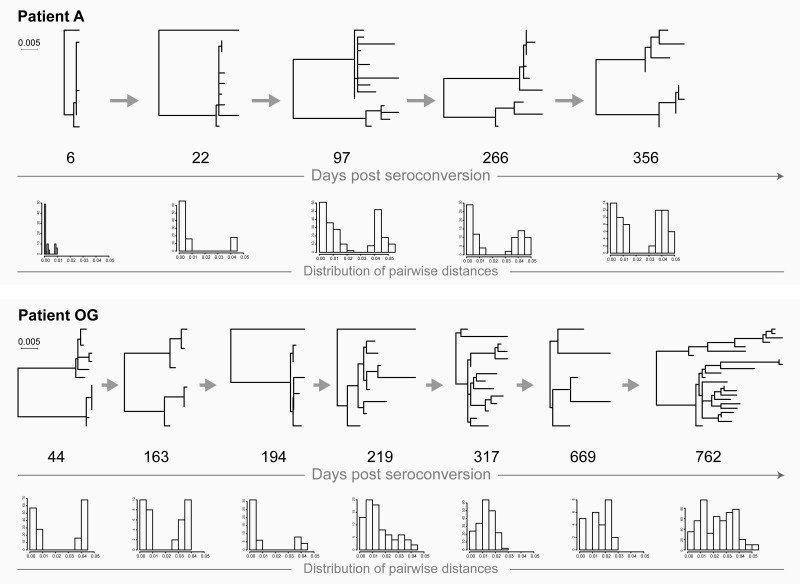
HIV transmission of multiple viral variants in patients A (acute HIV infection) and OG (recent infection). For explanation of phylogenetic trees, pairwise distances, and time points of sampling, please see Fig 1 legend.

**Fig 4:**
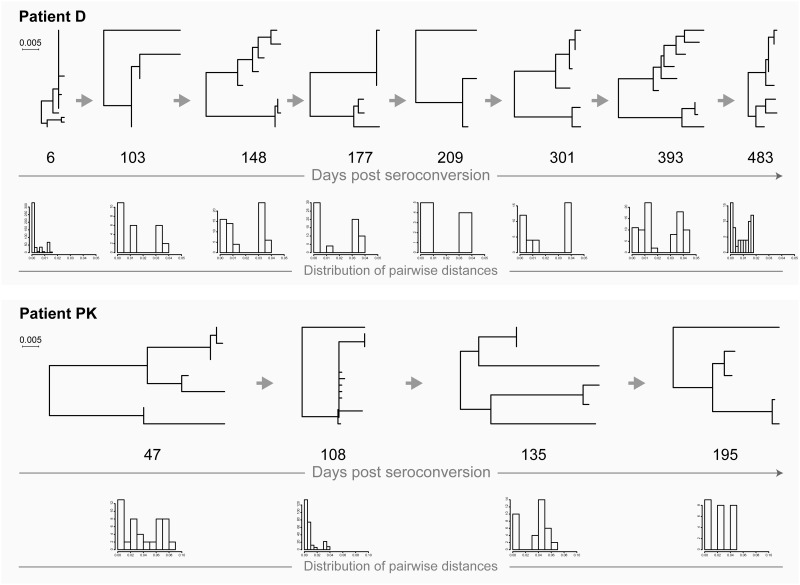
HIV transmission of multiple viral variants in patients D (acute HIV infection) and PK (recent infection). For explanation of phylogenetic trees, pairwise distances, and time points of sampling, please see Fig 1 legend.

This is an uncommon scenario of HIV transmission that is made evident by the specifics of the tree topology and the distribution of pairwise distances. In the phylogenetic tree, viral sequences representing intra-host HIV quasispecies form and can be identified as a distinct monophyletic clade with specific structure. At the earlier stage (e.g., within weeks or a few months after HIV transmission and seroconversion), the structure of the monophyletic clade includes multiple (i.e., two) sub-clusters with relatively low diversity within each sub-cluster (Fig 3: patient A at days 22 and 97, and patient OG up to day 194; Fig 4: patient D up to days 301/393, and patient PK up to days 108/135). The corresponding distribution of pairwise distances is characterized by two distinct peaks on the histogram indicating low levels of diversity within each sub-cluster and sizable pairwise diversity associated with pairwise distances between sub-clusters.

The typical tree topology upon transmission of multiple viral variants from the same source is transient, and therefore can be easily overlooked. The distinction between sub-clusters disappears over time, apparently due to *de novo* generated recombinants that can fill the gap between sub-clusters in the phylogenetic tree (as shown in our previous analysis [42]) and convergence of distinct peaks in the histogram with pairwise distances (Fig 3: patient OG at day 219 and later time points; Fig 4: patient D at day 483, and patient PK at day 195). Note that in patient A (Fig 3), virus sequences did not close the gap between sub-clusters by day 356.

### HIV super-infection

**Fig 5:**
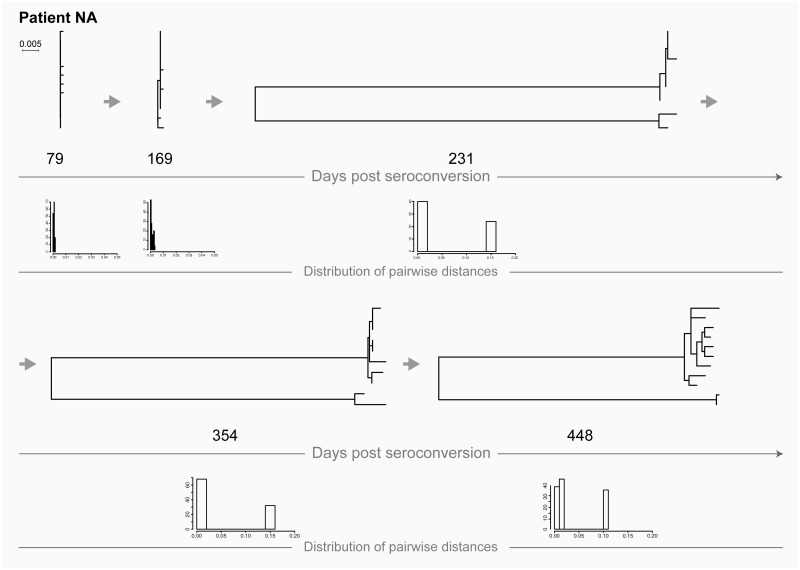
HIV transmission of single viral variant in patient NA (recent infection) followed by a super-infection. For explanation of phylogenetic trees, pairwise distances, and time points of sampling, please see Fig 1 legend.

Transmission of distinct HIVs can occur over the course of HIV infection. In contrast to a monophyletic clade, viral sequences representing super-infection fall into different parts of the inferred phylogenetic tree and are separated by HIV sequences from other patients. Upon HIV super-infection, long branches separate distinct viral variants. Multiple (i.e., two) peaks can be found on the histogram of pairwise distances reflecting distances within and between two viral variants (Fig 5: patient NA at days 231, 354, and 448). Note that at earlier time points, days 79 and 169, patient NA was infected with a single viral variant.

### Transmission of two founder viruses followed by a super-infection

**Fig 6:**
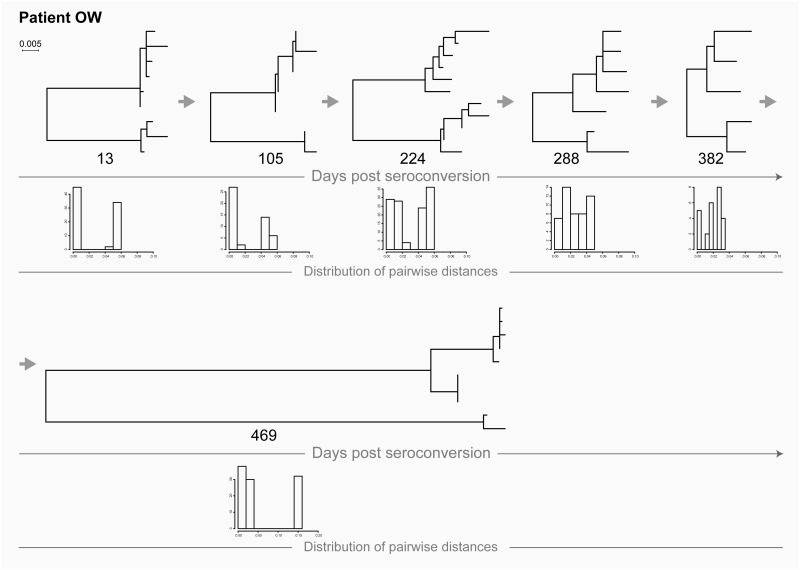
HIV transmission of multiple viral variants in patient OW (recent infection) followed by a super-infection. For explanation of phylogenetic trees, pairwise distances, and time points of sampling, please see Fig 1 legend.

As shown in Fig 6, patient OW was infected with two viral variants, which was evident from the inferred phylogenetic tree from days at earlier time points. Then, by day 469, this patient acquired a distinct HIV, constituting a super-infection with a distinct virus.

### Frequency of HIV transmission

The frequencies of different HIV transmissions within the *Tshedimoso* study cohort [38–40] are presented in Table 2.

**Table 2:** Sensitivity analysis. Frequency and rate of different types of HIV transmission in a cohort of 42 individuals with primary HIV-1C infection: phylogeny and pairwise distance analysis is compared with ratio *withinss* to *betweenss* threshold values.

*Types of HIV transmission*	Phylogeny & pairwise distances		Ratio *withinss* to *betweenss* threshold values									
			0.10		0.15		0.20		0.25		0.30	
	n	prop*	n	prop*	n	prop*	n	prop*	n	prop*	n	prop*
Single founder virus	33	0.79 (0.63–0.90)	35	0.83 (0.67–0.93)	34	0.81 (0.66–0.91)	31	0.74 (0.60–0.86)	28	0.67 (0.50–0.80)	21	0.50 (0.34–0.66)
Multiple viral variants from the same source partner	9	0.21 (0.10–0.37)	7	0.17 (0.07–0.31)	8	0.19 (0.09–0.34)	11	0.26 (0.14–0.42)	14	0.33 (0.20–0.50)	20	0.48 (0.32–0.64)
HIV super-infection	2	0.05 (0.01–0.17)	2	0.05 (0.01–0.17)	2	0.05 (0.01–0.17)	2	0.05 (0.01–0.17)	2	0.05 (0.01–0.17)	2	0.05 (0.01–0.17)
Multiple HIV variants from the same source partner followed by a super-infection	1	0.02 (0.001–0.14)	1	0.02 (0.001–0.14)	1	0.02 (0.001–0.14)	1	0.02 (0.001–0.14)	1	0.02 (0.001–0.14)	1	0.02 (0.001–0.14)

prop*: proportion (95% CI)

Based on phylogeny and pairwise distance analysis, transmission of a single viral variant was evident in 33 cases (79%; 95% CI 63–90%). Transmission of multiple viral variants from the same source was evident in 9 (21%; 95% CI 10–37%) cases. HIV-1 super-infection was identified in 2 cases (5%; 95% CI 1–17%). The estimated rate of HIV-1C super-infection is 3.9 per 100 person-years. Transmission of multiple viruses combined with HIV super-infection at a later time point was observed once. The population frequency of HIV transmission as multiple variants from the same source remains unclear and warrants further studies.

The frequency of multiple-variant transmission from the same source (21%; 95% CI 10–37%) appeared to be larger than the frequency of HIV superinfection (5%; 95% CI 1–17%), the difference reached statistical significance at the 5% level (p = 0.04; McNemar's test).

## Discussion

Multiplicity of HIV transmission has important implications for design and development of treatment and prevention strategies, and particularly for advancing HIV vaccine research. The extreme cases in multiplicity of HIV transmission, such as transmission of a single founder virus, or of substantially distinct multiple viral variants, are well defined. These cases can be detected relatively easily, e.g., by phylogenetic inference of viral sequences representing intra-host HIV quasispecies. However, HIV transmission of closely related multiple viral variants remains uncertain, and the criteria for identification of such multiplicity have not been defined, nor have its clinical or epidemiological relevance.

In this study we utilized HIV sequences representing intra-host viral quasispecies from a prospectively sampled cohort of 42 individuals in Botswana who were enrolled in a primary HIV-1C infection project, the *Tshedimoso* study [4, 38–45]. Viral sequences were generated by single-genome amplification and sequencing and spanned the V1C5 region of the HIV-1C *env* gp120. Transmission of at least two distinct HIV variants from the same source partner was demonstrated in 17% (7 of 42) of cases. The frequency of HIV super-infection was 5% (2 of 42) of cases, similar to the rate in MSM [56].

The identification of closely related multiple viral variants might be challenging. The transient nature of distinct sub-clusters requires sampling during the early stage of HIV infection. If the early time points of sampling are missed, the topology and branch length in the phylogenetic tree and distribution of pairwise distance might not be informative. Moreover, within a short time after transmission of multiple viral variants, the elevated branch lengths and the extended pairwise distances could be interpreted as evidence for an established (chronic) HIV infection, leading to a misclassified recent HIV infection. This phenomenon and sub-optimal sampling could complicate the use of viral diversity as a marker of HIV recency in population studies. However, knowledge of the pattern of multivariant HIV transmission from the same source and its frequency in different populations could help to refine the estimation of HIV recency. If analysis of HIV recency relies on viral diversity, an adjustment for transmission of multiple viral variants could improve accuracy and result in more precise estimation of HIV recency.

Sub-clustering of viral sequences could be defined by a topology of the inferred ML phylogenetic tree—presence of a monophyletic patient-specific lineage with sub-clusters, accompanied by a specific distribution of virus pairwise distances. However, such identification could be subjective, as the criteria for identification of sub-clusters are not well defined. To alleviate this problem and reduce subjectivity in identification of sub-clusters within the pool of HIV sequences representing intra-host viral quasispecies, we suggested a simple method based on the ratio withinss to betweenss. Our intention was to assess the extent to which the ratio withiness to betweeness can be used as a more objective surrogate for subjective interpretation of phylogeny plus pairwise distance distribution. We performed simulation studies (see Table 1), and found that the ratio values (withinss to betweenss) for both sub-clusters greater than zero and less than 0.20 are associated with high sensitivity (0.97) and moderate specificity (0.77), and were accompanied by acceptable positive and negative predictive values. A potential clonal expansion of viral variants, or bias in the sequencing system, may affect or even mislead identification of sub-clusters. This limitation of sub-clusters analysis provides a rationale for developing new methodologies and warrants further studies.

**Fig 7:**
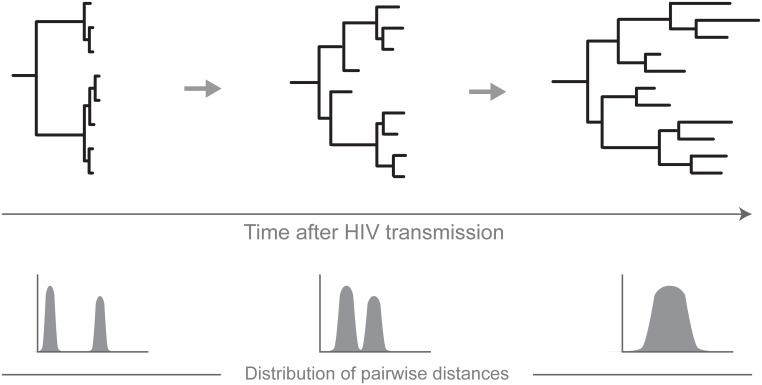
A simplistic model for HIV transmission of closely related multiple viral variants. The dynamics of phylogenetic trees and pairwise distances are presented over time of HIV infection.

A simplistic diagram in Fig 7 outlines the concept for transmission of multiple HIV variants from a single source partner. The diagram highlights only some key processes occurring during transmission of multiple viruses and does not intend to represent the complexity of HIV evolution. A monophyletic clade evident by a long, patient-specific branch separates viral sequences that represent intra-host HIV quasispecies from other patients’ sequences or reference sequences. At the early stage of HIV infection, the internal structure of the clade shows at least two distinct sub-clusters with low diversity of viral quasispecies within each sub-cluster. The histogram of pairwise distances has multiple (at least two) distinct peaks that could represent pairwise distances within and between sub-clusters. This is a transient phase. The duration of this phase could reflect complex virus-host interactions and is patient-specific. The branching pattern of the phylogenetic tree changes over time. The dynamic process of filling the gap between originally distinct sub-clusters deserves a separate investigation. Over time, the peaks of pairwise distances in the histogram could converge. The presented diagram does not reflect all possible scenarios, such as overlapping of peaks, or multiple peaks originating from alternative processes.

In summary, the results of this study suggest that upon HIV infection, transmission of closely related multiple viral variants from the same source can be distinguished from transmission of viral variants from different sources. The proposed simplistic model highlights the dynamics of multivariant HIV transmission from the same source. The frequency of this transmission in different populations needs to be addressed in future studies.

## Conclusions

Multiple HIV lineages transmitted from the same source produce a monophyletic clade in the inferred phylogenetic tree. Such a clade has transiently distinct sub-clusters in the early stage of HIV infection, and follows a predictable evolutionary pathway. Over time, the gap between initially distinct viral lineages fills in and initially distinct sub-clusters converge. Identification of cases with transmission of multiple viral lineages from the same source needs to be taken into account in cross-sectional estimation of HIV recency in epidemiological and population studies.
